# Gene Modulation with CRISPR-based Tools in Human iPSC-Cardiomyocytes

**DOI:** 10.1007/s12015-023-10506-4

**Published:** 2023-01-19

**Authors:** Julie Leann Han, Emilia Entcheva

**Affiliations:** Department of Biomedical Engineering, The George Washington University, 800 22nd St NW, Suite 5000, Washington, DC 20052 USA

**Keywords:** CRISPR, dCas9, CRISPRi, CRISPRa, Human iPSC-CMs, Gene modulation, Gene knockdown

## Abstract

Precise control of gene expression (knock-out, knock-in, knockdown or overexpression) is at the heart of functional genomics – an approach to dissect the contribution of a gene/protein to the system’s function. The development of a human in vitro system that can be patient-specific, induced pluripotent stem cells, iPSC, and the ability to obtain various cell types of interest, have empowered human disease modeling and therapeutic development. Scalable tools have been deployed for gene modulation in these cells and derivatives, including pharmacological means, DNA-based RNA interference and standard RNA interference (shRNA/siRNA). The CRISPR/Cas9 gene editing system, borrowed from bacteria and adopted for use in mammalian cells a decade ago, offers cell-specific genetic targeting and versatility. Outside genome editing, more subtle, time-resolved gene modulation is possible by using a catalytically “dead” Cas9 enzyme linked to an effector of gene transcription in combination with a guide RNA. The CRISPRi / CRISPRa (interference/activation) system evolved over the last decade as a scalable technology for performing functional genomics with libraries of gRNAs. Here, we review key developments of these approaches and their deployment in cardiovascular research. We discuss specific use with iPSC-cardiomyocytes and the challenges in further translation of these techniques.

**Graphical Abstract:**

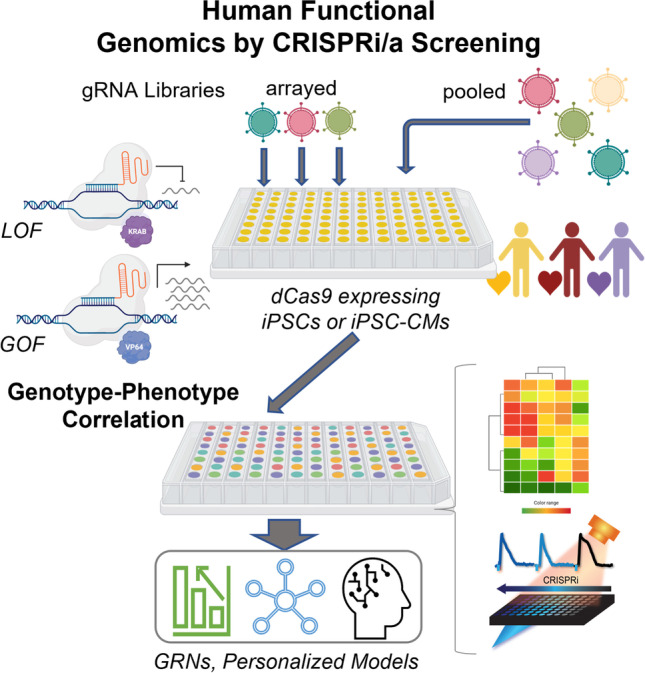

## Introduction

**Fig. 1:**
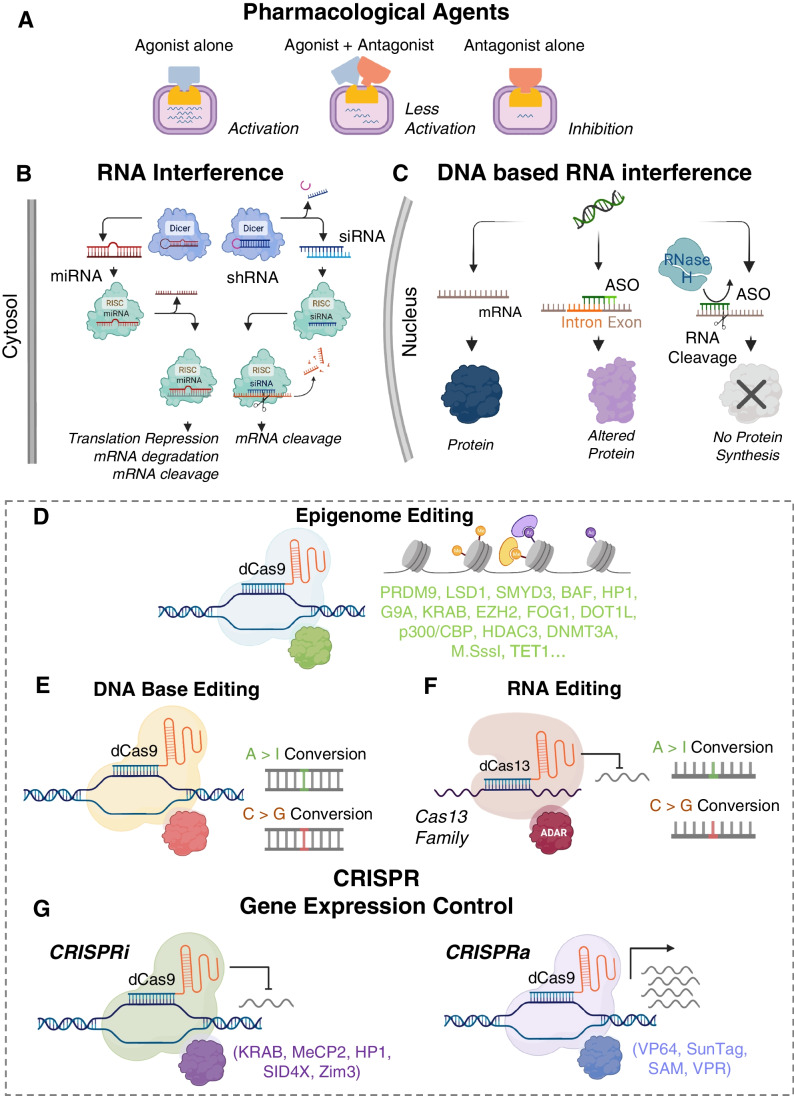
Approaches for gene modulation. (**A**) Traditional methods to control gene function involve pharmacological compounds. (**B**) Targeting miRNAs and shRNA/siRNA in the naturally occurring RNAi pathways has been used to control post-transcriptional gene expression. (**C**) Basic mechanism of antisense oligonucleotides for targeting protein expression. (**D**) Epigenome editing with CRISPR. (**E**) DNA base editing tools introduce single nucleotide edits to the DNA. (**F**) RNA base editors include cytosine and adenine versions. (**G**) CRISPR-based approaches for gene activation and interference. Biorender was used for parts of this figure

Gene modulation refers to the alteration of a gene for the mechanistic study of biology or toward therapeutic intervention (Fig. 1). Traditional methods to manipulate gene expression involve small molecules, DNA-binding agents, synthetic oligonucleotides or post-transcriptional modifications through RNAi. Over the last decade, developments of CRISPR technologies have expanded the toolkit to edit DNA or RNA with greater efficiency and precision for potential cardiovascular applications [1]. This review focuses on newer CRISPR-derived gene modulation methods, including those which do not induce permanent alterations to the genome. Human induced pluripotent stem cells (iPSCs) present a renewable supply of otherwise difficult to obtain human cell types, particularly in the study of cardiac [2], neurological [3], and metabolic diseases, showing great potential for drug development/screening, gene therapy, and regenerative medicine. The combination of these two scalable approaches, iPSCs and CRISPR-based gene modulation, has yielded high-throughput methods for genetic screens to uncover the molecular underpinnings of biological function and to address disease mechanisms. Such tools are poised to accelerate the development of new targeted therapeutics.

## Classic Methods for Gene Modulation

### Transcriptional Modulation by Small Molecules

Traditional drugs modulate the activity of a specific protein as agonists (activation), antagonists (inhibition) or by a mixed agonist–antagonist action, where they can have both activating and inhibiting properties, Fig. 1A. Small molecules can exert short or long-term manipulation of gene expression of a single or multiple genes. Despite recent advances, specificity is hard to achieve and off-target interactions are a major drawback as small molecules interact with unintended targets, which may cause pharmacological toxic events [4].

Conventional approaches toward cardiovascular drugs have focused on accessible targets (at the membrane surface) and signaling cascades, such as G-protein-coupled receptors (GPCRs); blocking of neurohormones (catecholamines, angiotensin, aldosterone); ion channels blockers; and targets related to pathological load (vasodilators and diuretics) [5]. Human iPSC-cardiomyocytes express the key cardiac ion channels and have relevant GPCR machinery [6], therefore, they represent a more physiologically-relevant alternative to heterologous systems for the testing of new small molecules [7]. When derived from patients carrying certain ion channel mutations, these experimental models provide valuable information about the efficacy and safety of newly developed drugs at the pre-clinical stage. For example, McKeithan and colleagues used high-throughput screening methods in human iPSC-CMs from healthy controls and patients with long-QT syndrome type 3 (LQT3) having a mutation in the SCN5a sodium channel [8]. Through medicinal chemistry optimizations, they derived new mexiletine derivatives with better specificity and efficacy to target the late sodium current that is the culprit in LQT3.

Additionally, protein kinases, such as Ca^2+^-calmodulin-dependent protein kinase (CaMK), phosphoinositide 3-kinase (PI3K), mitogen-activated protein kinase (MAPK), mechanistic target of rapamycin (mTOR), protein kinase A (PKA), and protein kinase C (PKC) have been targeted for pharmacological treatment of cardiac diseases, including heart failure, cardiomyopathies, myocardial infarction and arrhythmias [9, 10]. Recent studies demonstrate convincingly the utility of human iPSC-CMs in testing MAPK inhibitors in combination therapy when immunosuppressants are also used [11] and in large-scale screening of kinase inhibitors [12]. The highly parallel nature of these assays and the multi-parametric readouts enable mechanistic insights and can lead to more reliable predictors of drug action.

Another pharmaceutical approach for pathological cardiomyopathies is to regulate nuclear gene transcription by using small molecules to target epigenetic modifiers, e.g. histone acetyltransferases (HATs) and histone deacetylases (HDACs). HATs and HDACs are master regulators that activate gene transcription by acetylating nucleosomal histones and relaxing the chromatin structure, or inhibit transcription by deacetylation nucleosomal histones, respectively [13]. For example, hydroxamic acid trichostatin A (TSA), an HDAC inhibitor, is a potent repressor of cardiac hypertrophy and has been shown to regulate multiple genes within the hypertrophic cascade [14]. Over 500 clinical trials are under way with HDAC inhibitors for various applications, mostly cancer-related [15, 16], and the potential cardiotoxic effects of HDAC inhibitors have been tested in pre-clinical studies with human iPSC-CMs [17]. Overall, gene modulation by small molecules is attractive due to its translational potential, yet specificity of action is hard to achieve.

### RNA Interference

A key approach to post-transcriptional gene modulation is RNA interference (RNAi), Fig. 1B. RNAi mirrors traditional drug therapies as it is used to silence the gene and encoded protein for a defined target [18]. In a cell, RNAi occurs naturally via microRNAs (miRNAs) and other noncoding RNAs to regulate gene expression. Inhibitory RNAs can be designed to mimic miRNA, small interfering RNAs (siRNAs), or short-hairpin RNAs (shRNAs), by using complements to their targets. siRNAs, chemically synthesized double-stranded RNAs, exert gene silencing by loading onto RNA-induced silencing complex (RISC). This leads to the endonuclease cleavage of the passenger strand by Argonaute-2 and consequent gene inactivation. shRNAs are stem-loop structures that can be expressed in vector systems such as plasmids or viral vectors. They mimic precursor miRNAs and are exported from the nucleus to the cytoplasm by Exportin-5, a nuclear membrane protein, and cleaved by the Dicer complex in the cytoplasm [18]. Small RNA duplexes are produced, developing into mature double-stranded siRNA, and finally leading to mRNA degradation by RISC and Argonaute-2 processing.

In contrast to siRNA, which post-transcriptionally targets a specific gene, miRNAs have broader action and typically inhibit gene expression of multiple mRNAs. miRNAs are transcribed in the nucleus to give rise to primary miRNA (pri-miRNA). The pri-mRNA is cleaved to form a precursor miRNA and processed by Exportin-5 and the Dicer complex as described above. After the passenger strand is discarded, the mature single-stranded miRNA can target mRNAs through partial complementary base pairing, leading to target gene silencing via translational repression, degradation, and/or cleavage [19]. miRNAs theoretically have broader therapeutic applications as they can target complex multigenic diseases, e.g. cancers, neurodegenerative disorders, and cardiovascular disease [20]. In contrast, siRNAs are best suited to treating monogenic diseases [21, 22], although some clinicals studies have reported the use of siRNAs to target cancers and viral infection. Human iPSC-CMs are an important experimental model in the testing of siRNAs and miRNAs for therapeutic purposes. For example, screening in iPSC-CMs led to the discovery of miRNAs involved in the biogenesis of atrial natriuretic peptide – a key biomarker in hypertension and heart failure and a potential therapeutic target [23].

In 2004, the first siRNA therapeutic reached clinical trials, while the first miRNA clinical trial did not begin until in 2013 [24]. The slower progress of miRNA drug development may be attributed to its uncertain mechanisms of action and specificity. Currently, there are three FDA approved siRNA drugs and seven in late stages of Phase 3 clinical trials [25]. In 2018, patisiran was the first FDA approved siRNA-based therapeutic to enter the pharmaceutical market. Patisiran was developed for the treatment of transthyretin (TTR)amyloidosis that can lead to severe congestive heart failure. It was shown to improve cardiac structure and function in patients with cardiomyopathy [25, 26]. Vutrisiran is another investigational siRNA-based treatment targeting TTR in Phase 3 clinical trials for the treatment of amyloidosis with cardiomyopathy. It is a second generation therapeutic with chemical modifications, introduced to increase potency and metabolic stability. Inclisiran is a second-generation siRNA conjugate that inhibits PCSK9 for the treatment of atherosclerotic cardiovascular disease by reducing LDL-C levels and is under review for approval [25]. Overall, RNAi (siRNAs and miRNAs) has found broad applications in cardiovascular research, potential cardiovascular stem cell therapy, and the identification of protein coding genes and non-coding RNAs in cardiology [26].

Despite the therapeutic potential of siRNAs and miRNAs, they face notable challenges, such as proper delivery and uptake to target site, low bioavailability, rapid clearance, variability amongst tissue types, activation of immunogenic responses, and degradation by nucleases present in the plasma, tissues and cytoplasm [27]. Chemical modifications and optimized delivery have been applied to improve their pharmacokinetics, pharmacodynamics, and safety profile [27]. While these strategies have improved the stability of siRNA, in some cases, they have also increased toxicity and reduced gene silencing [28]. siRNA can only tolerate limited chemical modifications without impairing the activity of RISC [29].

### DNA-based RNA Interference with Antisense Oligonucleotides

Unlike siRNA, which are double stranded RNA molecules, antisense oligonucleotides (ASOs) are short synthetic single-stranded DNA oligomers around 10–30 nucleotides long, that can differentially regulate gene expression, Fig. 1C [30]. ASOs have less toxicity and lower off-target effects on activating the host immune system, compared to siRNAs, because of their higher tolerance to and wider range of possible chemical alterations [31]. ASOs can be synthesized to either downregulate a molecular target or to modulate alternative splicing. To induce gene silencing, the designed antisense strand prompts RNase H endonuclease activity that cleaves RNA–DNA hybrids, significantly reducing target gene translation [32]. ASOs can also be designed to regulate RNA splicing [30], i.e. the removing of introns from the initial transcription product of the DNA and joining the protein-coding regions (exons) to form a continuous RNA molecule. Alternative splicing is regulated to silence or enhance target proteins that can produce differing variants with distinctive functions. ASOs can regulate splicing by binding to the mRNA precursor to block the binding of splicing factors, changing the original splicing pattern, and activating a new splicing site, to forcibly include desired exons [30]. Other ASO-driven mechanisms include altering the splicing process (splice-switching), and sterically obstructing ribosomal activity [32].

There are multiple FDA-approved ASO-mediated therapies and some still in ongoing clinical trials. In 1998, formivirsen became one of the first FDA approved ASO drugs, used for the treatment of cytomegalovirus. In 2013, mipomersen was the first FDA approved ASO for cardiovascular indication – treatment of familial hypercholesterolemia via suppression of Apolipoprotein B, a major determinant of cardiovascular risk [26]. In 2016, eteplirsen was approved to treat Duchenne muscle dystrophy (DMD) [33]. Some of the drawbacks of using ASOs in the clinic are similar to RNAi in that they are also subject to degradation by nucleases, concerns about uniform delivery to tissues, imperfect binding to the target mRNA, and off-target effects and toxicity [26]. Improved ASOs have been developed by the introduction of phosphorothioate linkages to replace the phosphodiester bonds between the nucleotides that form the backbone to improve stability, increase cellular uptake, and prevent degradation [26, 27].

## Evolution of the CRISPR Technology

Initially identified as part of the natural antiviral defense system of bacteria and archaea, CRISPR are short nucleotide repeats that are used to detect and destroy DNA during infection. CRISPR RNA (crRNA) functions to guide Cas proteins to the invading nucleic acid in order to degrade the nucleic acid during the innate bacterial defensive pathway. Cas9 enzymes combined with CRISPR sequences form the foundation of the CRISPR-Cas9 technology that has transformed genome editing. In 2011, Charpentier and colleagues discovered that trans-activating CRISPR RNA (tracrRNA) is necessary for the maturation of crRNA in studies with *S. pyogenes* [34]. Around the same time, Siksnys and colleagues cloned the entire CRISPR-Cas9 locus from *S. thermophilus* in E. Coli [35]. They also purified Cas9 with crRNA and were among the first to characterize Cas9’s mechanism of action [36]. They reported that Cas9 could be directed to different target sites by manipulating the sequence of the crRNA and identified that the protospacer adjacent motif (PAM) sequence was necessary for initial DNA binding and cleavage by the Cas nuclease [36]. Similar findings were described by Charpentier and Doudna [37], where they showed that the crRNA and tracRNA could be merged to create a single, synthetic guide RNA (gRNA) that interacts with the DNA target and with Cas9, further simplifying the technology. These discoveries catapulted efforts to apply the technology to edit genomes. Work in Zhang’s group at MIT [38] and Church’s group at Harvard [39] led to the application of CRISPR gene editing in mammalian cells.

### Gene Editing with CRISPR-Cas9 Including Prime Editing

Cas proteins, along with transcription activator like nucleases (TALENs) and zinc finger nucleases (ZFNs) are site-specific nucleases that enable genetic modifications by inducing double strand breaks (DSBs) at target locations in the genome. All have broad applications for experimental biology and therapeutic purposes, but CRISPR/Cas9 is superior in accuracy and specificity in that it only requires a single protein domain for RNA-guided DNA recognition and cleavage; whereas ZFNs and TALENs require two individually synthesized protein domains. These tools, in their classic form, rely on the activation of two DNA-repair machinery pathways: non-homologous end joining (NHEJ) and homology directed repair (HDR). The highly error-prone NHEJ pathway joins the fragmented ends together, which often introduces insertions and deletions (indels) that result in frameshift mutations and subsequent gene knockout. The HDR pathway is a precise repair mechanism that allows directed recombination between a DNA donor template and the cut DNA site to correct the DSB. Consequently, HDR can be used to introduce specific mutations or transgenes into the genome [40]. However, many human cell types, including human iPSC-CMs, are relatively incompetent in carrying out HDR with high efficiency.

Conveniently, CRISPR prime editing bypasses the need for HDR and has been shown effective in a broad range of cell types [41]. It uses a Cas9 nickase (a mutant Cas9 that can induce single-strand “nicks”/cuts), fused to an engineered reverse transcriptase (to induce target-primed reverse transcription (TPRT) and a prime editing gRNA (pegRNA) to introduce new sequence information into the genome to a locus-specific region without the need of a donor template [41]. Although initial prime editing efficiencies were low, modifications have improved the technology to exhibit better stability and more efficient pegRNA design [42]. Prime editing generally performs shorter edits of about 20 bps, but can introduce larger genomic deletions on both sides of the target DNA [43]. CRISPR prime editing is a broadly useful genome editing technology for the investigation of complex genetic changes. Another CRISPR-derived approach that does not introduce DSBs, similar to prime editing, involves the catalytically inactive dCas9 and is discussed below as part of reversible gene modulation methods.

CRISPR/Cas9 has already been applied for in vivo studies since its discovery in 2011. *S. pyogenes* Cas9 (4.1 kbps) is still the most widely used protein for genome editing. A limiting factor for its translation in the clinic has been the delivery of CRISPR-Cas9 components into cells. Adeno-associated viruses (AAVs) are the most often used viral vectors for clinical and in vivo studies because they induce mild immune response in humans, and do not get integrated into the host’s genome. AAVs have been FDA approved for the treatment of a variety of diseases. Unfortunately, the maximum packaging capacity of AAVs is about 4.7 kbps, which leaves little room for the addition of gRNAs or regulatory factors for CRISPR gene editing studies in vivo. One method has been to directly inject Cas9 and gRNA protein or mRNA into embryos to develop transgenic animals, as for example done for cardiac applications in [44–46], Table 1. Because of the limitations of packaging Cas9, AAV packaged gRNAs have been delivered to Cas9 expressing transgenic mouse models [47, 48]. Some studies have separately packaged Cas9 and gRNAs into AAVs for intravenous delivery [45, 49–53]. Despite the packaging limit of AAVs, successful singular delivery of Cas9 and gRNA together via AAV transduction has been reported for the correction of DMD [54, 55] and correction of faulty RyR2 to prevent ventricular arrhythmias [56]. Alternative methods of co-delivery have used liposomes [57] and adenovirus [58, 59], despite the limitations of clinical translation due to adverse immune response and systemic toxicity of adenoviruses. More recent methods to employ smaller Cas9 variants, such as *S. aureus* (SaCas9) or *S. thermophilus* (St1Cas9) are being pursued to circumvent these limitations. Although CRISPR/Cas9 is an invaluable method for disease modelling, its potential use in vivo requires further exploration to overcome the limitations of the current delivery methods and the off-target events observed [45, 52, 60]. For example, AAV-CRISPR constructs triggered immunogenic response in adult mice but not in neonatal animals; silencing of Cas9 and the gRNAs was observed within six months, and some limited unintended genetic modifications were documented while applying the technology to correct DMD mutations in muscle [52]. When the CRISPR-Cas9 system was delivered in patient-derived iPS lines to correct heterozygous *MYBPC3* mutations, responsible for hypertrophic cardiomyopathy, it generated indel-inducing NHEJ repairs in over half of the targeted lines. In contrast to iPS cells, when CRISPR-Cas9 editing of *MYBPC3* was done in human embryos, targeting efficiency was much higher, and HDR was the predominant repair mechanism [60].

**Table 1:** In vivo gene editing with CRISPR/Cas9 – select cardiac applications

Disease type	Model organism	Gene target editing	Disease	References
Cardiomyopathies, Pro-arrhythmic phenotypes	Human	*MYBPC3 KI*	Hypertrophic cardiomyopathy	[60]
	Mouse	*Myh6 KO*	Cardiomyopathy disease modelling	[47]
		*PLN KO*	Heart Failure	[44]
		*Prkag2 KI*	Wolff-Parkinson-White sydrome	[45]
		*Myh6, Sav1, Tbx20 KO*	Dilated Cardiomyopathy, arrhythmia	[48]
		*Ryr2 KI*	Ventricular Tachycardia	[56]
		*Idua KI*	Mucopolysaccharidosis type I	[57]
	Zebrafish	*Kcnj8, Sur2, Abcc9 KI*	Cantú Syndrome	[46]
Cardiovascular Screening	Zebrafish	*flt4, ccbe1, vegfab KO*	Disease Modelling	[61]
Duchenne Muscular Dystrophy (DMD)	Mouse	Ex23 DMD *KO*	Ex23 DMD	[49, 50, 54, 58]
		Ex52 and Ex53 DMD *KO,* Ex53 DMD *KI*	ΔEx52 and ΔEx53 DMD	[55]
		Ex44 DMD *KO*	ΔEx44 DMD	[51]
		Ex23 DMD *KO*	ΔEx23 DMD long-term effects of treatment	[52]
	Rabbit	Ex51 DMD *KO*	ΔEx51 DMD animal model	[62]
	Monkey	Ex4 and Ex46 DMD *KO*	ΔEx4 and ΔEx46 DMD animal model	[63]
	Canine	Ex51 DMD *KO*	ΔEx50 DMD	[53]

### Gene Editing with CRISPR/Cas9 in Human iPSC

Genome editing in iPSCs can be used to dissect genetic, molecular, and cellular mechanisms particularly in cardiac, neurodegenerative, and metabolic diseases – as it is otherwise difficult to obtain these cell types and recapitulate disease phenotypes in vitro. CRISPR-based editing allows the generation of isogenic controls, where a disease-associated mutation is introduced or corrected to reveal its impact on an identical genetic background in disease modelling. In cardiac applications, disease-associated mutations have been corrected in hiPSC-CMs by CRISPR/Cas9 as potential methods to treat cardiomyopathies [2, 64–66]. In vitro, Cas9 was also applied to knockout Nav1.5 to model LQT3 [67], or to knock-in mutant CACNA1C for disease modelling [68], as well as for iPSC-based genome-wide screens [69–72], Table 2. The combination of these technologies enables the quantification of the contributory role of each genetic alteration in the context of disease and regenerative medicine.

**Table 2:** Cardiac gene editing applications with CRISPR/Cas9 in human iPS cell lines

	Disease Type	Gene Target	Disease	Reference
Cardiac applications	Cardiomyopathy	*PRKAG2 KI*	Familial Wolf-Parkinson-White Syndrome	[64, 73]
		*MYH7* variants *KO*	Hypertrophic cardiomyopathy model	[74]
		*TNNT2 KO*	Ventricular Arrhythmogenesis model	[65]
	LQTS	*KCNH2 KI*	LQT2 model	[66]
		*CACNA1C KO*	LQT8 model	[68]
		*SCN5A KO*	LQT3 model	[67]
	DMD	DMD *KO*	Duchenne muscular dystrophy correction	[51, 75]
	Disease-Modelling	Multi-gene *KO*		[76, 77]
iPSCs	Screens	Multi-gene *KO*		[69–72]

The usage of patient-derived hiPSCs further allows the interrogation of common and rare genetic variants across distinct genetic backgrounds for more inclusive complex models of genetic disorders [78, 79]. This paves a path towards precision medicine and the potential for patient specific drug screening, and the ability to predict responses to clinical treatment during the pre-clinical in vitro stages [7]. Despite the potential of iPSC technology, there are some outstanding challenges, as for example the reported variability between iPSC lines which may interfere with the precise characterization of genetic variants [80]. iPSC derivation and differentiation involve procedures, for which small variations at each step can significantly impact the overall phenotype. Patient-derived iPSCs of differing genetic backgrounds largely contribute to the functionality of iPSC-derived specialized cells (e.g. cardiomyocytes), as do non-genetic factors, such as culture conditions, passage and sex [81]. An individual donor’s genetic makeup combined with different iPSC derivation protocols may also impact their epigenetics, thus affecting pluripotency and the capacity to differentiate [82]. Furthermore, recent studies achieving high expression of CRISPR/Cas9 have revealed that the introduction of DSBs by Cas9 in iPS cells is toxic with p53 dependence [69]. This presents greater challenges for successful and homogeneous genome editing in these cells by CRISPR-Cas9, compared to genome editing in embryos or other cell types [60]. The ability to deploy some of the gene modulation approaches in post-differentiated cells may help circumvent these concerns and still benefit from isogenic pairs to identify how a particular genetic variant may be involved in cardiac, neurodegenerative, and metabolic disease and development.

## Gene Modulation with CRISPR-based Methods

### CRISPR for Epigenetic Control

Beyond gene editing (knockout/knock-in) studies, the CRISPR technology has been adopted for gene modulation methods without double strand cuts in the DNA and can be deployed in post-mitotic cells in a time-resolved manner. These include gene activation (CRISPRa), gene inhibition/interference (CRISPRi), epigenome editing, DNA base editing and RNA base editing, Fig. 1D–G. These methods use a catalytically dead Cas9 (dCas9) with preserved site-targeting ability. When combined with proper effectors (transcription factors) and gRNAs, instead of generating DSBs, dCas9 can be applied for activation or inhibition at a site. CRISPRi/a rely on action via epigenetic regulators involved in DNA methylation, histone acetylation, or histone methylation. Therefore, there is considerable overlap between CRISPRi/a and epigenetic engineering techniques. For instance, Krüppel-associated box (KRAB) for CRISPRi domain induces histone methylation for gene inactivation. Conversely, effectors used in CRISPRa, such as VP64 [83], VPR [84], Suntag [85], and Synergistic activation mediator (SAM) [86] induce epigenetic changes, e.g. histone acetylation, to activate genes [87–90]. These transcription factors have also been applied towards epigenetic engineering studies [87, 88, 90]. Epigenetic control is a powerful way to modulate genes by introducing chemical and topological changes in the DNA organization. These factors contribute to the epigenetic state of the cell and modulate gene expression, cell fate, and ultimately cellular phenotype. Numerous studies have shown how epigenetic modifications can affect cardiac development and disease [1, 16, 91]. However, until recently, we have not had the tools to study how each epigenetic feature can contribute to changes in cardiac function.

For locus specific editing of chromatin marks, dCas9 enzymes have been used to recruit various epigenetic effectors, Fig. 1D. Methylation at H3K4 revealed upregulation of transcriptional activity and methylation of H3K9me, H3K27me and H3K79me allowed for gene repression [88]. Acetylation of H3K27 also has been associated with active promoters and enhancers [88]. Recently, Nunez et al. demonstrated CRISPRoff – a tool for programmable epigenetic memory based on DNA methylation, that can induce heritable gene inhibition [92]. By combining differential epigenetic modifiers and widely targeting dCas9, it is possible to identify how changes in the chromatin machinery within a specific DNA region affect gene expression. For example, dCas9 fused to histone demethylase LSD1 can be used to specifically define and target enhancers [93]. These tools help identify the epigenetic manipulations that contribute to biological function. Unfortunately, chromatin editing leads to modest gene expression changes when compared to CRISPRi/a gene modulation [84]. Thus, to achieve long-term, significant changes to the genome, there is still a need to further develop chromatin editors.

### Gene Modulation by DNA Base Editing

Base editing is a recent approach for gene modulation and some of its variants are derivatives of CRISPR/dCas9, Fig. 1E. Base editing strategies capitalize on the specificity of CRISPR but circumvent some of the limitations using Cas9 nuclease, namely the low efficiency of the HDR machinery, the need for donor DNA, the toxicity due to DSBs [69], and the inability to use CRISPR/Cas9 for postmitotic cells. Base editors were developed to allow targeted point mutation of a single DNA base without causing DSBs or needing donor templates [94, 95]. Current methods include a cytosine base editor (catalyzes C > T transition on PAM strand, or G > A transition on target strand) or adenosine base editor (catalyzes A > G transition on PAM strand, or T > C transition on target strand), fused to dCas9 [1, 95]. To create the first base editor, Komor et al. utilized a naturally occurring cytidine deaminase enzyme, APOBEC1, fused to dCas9 [94]. When a gRNA directs the APOBEC1-dCas9 fusion protein (BE1) to the target site, the deaminase converts C to uracil (U), which has base-pairing properties of thymine (T), within a window of approximately five nucleotides. They further modified BE1 to facilitate the removal U from DNA in cells and initiate base excision repair (BE2). Then, resolved the U:G mismatches into U:A and T:A pairs (BE3), significantly improving the base editing efficiency, with less than 1% indel formations [94].

Because naturally occurring adenosine deaminases that act on single-stranded DNA are not known, Guadelli et al., transformed a bacterial enzyme TadA, which naturally converts A > I/G in RNA, to welcome a DNA substrate to employ in mammalian cells and called it ABE7.10 [96]. Newer versions of base editors, such as BE4 [97] or ABE8 [98], offer improved efficiency in mammalian cells, allowing to screen for base-edited genetic variants [99] and applications *in vivo* [59, 100, 101]. Adenoviral delivery of BE3 has been used to correct *PCSK9* to treat atherosclerotic cardiovascular disease [59, 100, 101], and BE3 was reported to have more precise editing than Cas9 with no off-target events nor chromosomal translocations. Verve Therapeutics has recently obtained regulatory clearance to use this in humans. Before that, Beam Therapeutics received US approval to begin clinical trials of base editing therapy for sickle cell disease. Despite these advances, because spCas9-BE3 (5.1 kb) exceeds the AAV packaging capacity, strategies are still needed to develop smaller, functioning proteins to translate safely into the clinic. Separately, the deaminase is always active, which potentiates off-target effects by inducing bystander edits. In post-mitotic cells, base editing has been shown to be only about 10% efficient, still an improvement compared to HDR [102].

### RNA Base Editing by dCas13

RNA-based editing uses the Cas13 family of enzymes and its catalytically inactive counterparts (dCas13) to act on RNA rather than DNA, Fig. 1F. The protein and RNA components are similar to CRISPR/Cas9 and are programmable to cleave RNA or make RNA base edits by RNA Editing for Programmable A to I Replacement, version 2 (REPAIRv2) or RNA Editing for Specific C-to-U Exchange (RESCUE) [103, 104]. REPAIRv2 comprised an inactive/dead *Prevotella sep.* P5-125 Cas13b (dPspCas13b) and a mutant ADAR2 deaminase domain to induce A > I edits [103]. By protein engineering and directed evolution, ADAR2 was fused to dRanCas13b and termed RESCUE to allow for C > U edits [103, 104]. RNA editing holds several advantages over DNA editing, e.g. reversibility and minimal cytotoxicity due to the action only being applied post-transcriptionally. RNA editing can also be applied to non-dividing cells, e.g. cardiomyocytes, because it does not rely on endogenous repair mechanism such as NHEJ and HDR.

Many disorders are caused by splice variants that lead to gain-of-function mutations, loss-of-function mutations, or an accumulation of repeats containing transcripts leading to abnormal RNA foci in the nucleus. RNA base editing is a suitable approach to alter splicing variants, disrupt RNA–RNA base pairing, or eliminate toxic RNA as potential strategies to overcome disease. The approach can be a useful tool to treat viral infections or disorders that alter protein function caused by signal transduction [103]. The Cas13 family of enzymes are small and can be packaged into AAVs, allowing use in translational medicine [105].

### CRISPRi/CRISPRa

**Fig. 2:**
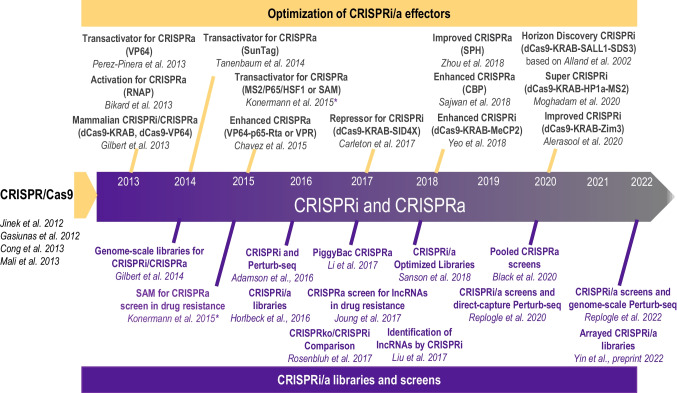
Timeline of CRISPRa and CRISPRi advancements. Developments of CRISPR-based tools for gene activation (CRISPRa) and inference (CRISPRi), and their expansion into genome-scale gRNA libraries and screens

Similar to epigenome editing, most CRISPRi and CRISPRa technologies use a subset of the transcription factors to allow for gene activation or inhibition, Fig. 1G. The evolution of CRISPRi/a methods is shown in Fig. 2. It was observed in bacterial and mammalian cells that dCas9 alone could still target the transcription sites of genes and block transcription without altering the DNA, termed CRISPR interference (CRISPRi) [106]. CRISPRi provided an alternative or a complementary approach for gene knockdown to avoid cellular toxicity and increase specificity to alter transcription while preserving the genetic structure [3, 107]. To improve the efficacy of gene silencing by CRISPRi, additional transcription repressors were explored [106]. dCas9-KRAB was found to exert a fivefold suppression compared to a twofold suppression by dCas9 only [108]. The KRAB domain interacts with KAP1, which recruits inhibitory factors heterochromatin protein 1 (HP1), histone deacetylases, and SETDB1, to suppress transcription [109, 110]. As suppression mediated by dCas9-KRAB was observed to vary from gene to gene [108], Carleton et al. investigated combinatorial relationships between enhancers and found that the addition of the SID domain to dCas9-KRAB improved knockdown [111]. The SID domain recruits histone deacetylases 1 and 2 (HDAC1/2) and removes histone acetylation markers associated with activation [112]. Although the dCas9-SID fusion had been employed as an enhancer [86], previous studies with TALENS showed the SID domain could be used to repress transcription [113]. Yeo et al. engineered and screened a more effective transcriptional repressor [114]—MeCP2, which binds to a different set of transcriptional regulators. They found that KRAB-MeCP2 was the most potent across all targets tested and exhibited improved repression compared to dCas9-KRAB [114]. Further developments led to a super CRISPRi where two transcriptional repressors were fused – heterochromatin protein 1 (HP1a) and KRAB with the MS2 coat protein and superior repression of genes of interest was reported in vivo compared to the previously published MeCP2 [115]. HP1a protein contains a chromodomain and a CS domain that interact with methylated histone H3 lysine 9 (H3K9) and H3K9-specific histone methylases [116]. Most recently, Alerasool et al. reported on various KRAB domains for improved suppression and found that KRAB-ZIM3 was consistently more potent than KOX1-KRAB and KOX1-KRAB-MeCP2 [117]. Additionally, KRAB-ZIM3 is smaller than the KOX1-KRAB-MeCP2, advantageous for viral delivery, and is less sensitive to gRNA selection than previously developed systems [117]. The commercially available Horizon Discovery’s CRISPRmod CRISPRi system uses dCas9 fused with SALL1 and SDS3 to inhibit gene transcription by recruiting proteins involved in chromatin remodeling and gene silencing. They observed that the dCas9-SALL1-SDS3 was equally specific compared to dCas9-KRAB; however was more potent in target gene repression, based on an earlier study [112]. While CRISPRi systems have been adopted for single target and multiplexed gene silencing [118], these approaches are not always consistent [119], and require further optimization.

The fusion of dCas to gene-regulatory proteins is also deployed for upregulation of genes (termed CRISPRa), Figs. 1G, and 2. Methods for gene activation were initially published in 2013 by using VP64 [83] and RNA polymerase (RNAP) [120]. VP64 is a strong transactivation domain that recruits the HAT p300 and activation complexes, causing DNA methylation and increased chromatin accessibility and activation of genes [89]. dCas9-VP64 is the first generation CRISPRa and achieves modest levels of activation. Effectors for CRISPRi, developed later, such as SunTag [85], SAM [86], and VPR [84], Fig. 2, all exhibit enhanced activation of genes, compared to the initially developed dCas9-VP64 [83], and provide flexible alternatives for experimental design. SunTag is an activation system that utilizes a scaffold of multiple VP64 activators to the dCas9 to parallelize the action of the transcriptional machinery to be recruited per gene, demonstrating a stronger activation with a single gRNA compared to dCas9-VP64 [85]. SunTag outperforms first generation activators but exhibits lower activation levels than SAM. SAM utilizes the dCas9-VP64 fusion protein and engineered sgRNAs to increase transcription. The engineering involves modifying portions of the gRNA into MS2-targeting aptamers [86], which then recruit additional activation domains; heat-shock factor 1 (HSF1) and the p65 subunit of the NF-κB complex. SAM has been shown to exhibit the most efficient levels of activation for single-gene targets. VPR – VP64/p65/Rta [84] was designed to activate transcription using three potent effectors—VP64, p64 and Rta—fused to dCas9. Despite its lower activation efficiencies compared to SAM, VPR is attractive for delivery because it offers a single-component system. For multiplexed gene regulation, SAM, SunTag and VPR have shown similar activation capacity. Newer hybrid methods for gene activation are emerging, such as SunTag-p65-HSF1 (SPH) [121], which replaces the VP64 domain in SunTag with P65-HSF activation domains from SAM. The resultant hybrid yields two- to three-fold improved activation efficiency compared to SAM, SunTag and VPR. More recently, a group designed an effector based on a HAT domain (CBP) that also outperformed SAM [122].

### Use of CRISPRi/CRISPRa in Cardiac Applications

CRISPRi/a can be used to identify key genes in cardiac development and disease in vivo and in vitro*,* Table 3. Friedman et al. [123] applied CRISPRi and conducted extensive single-cell RNA sequencing analysis during iPSC-CM differentiation to reveal gene networks for a more adult-like phenotype. By using an inducible CRISPRi system, Eskildsen et al. [124] identified that MESP1, a critical transcription factor in early cardiac development, is also necessary for vascular progenitor specification. Neiman et al. [125] observed active involvement of integrins (alpha5 subunit) in cardiac stem cell differentiation and contractility, suggesting their role in early stages of mesoderm specification and their downregulation upon cardiomyocyte differentiation. Schoger et al. [126, 127] created homozygous CRISPRi and CRISPRa hiPSC lines. Importantly, in these cell lines, CRISPRi and CRISPRa did not alter cells’ ability to differentiate into the three germ layers, and to produce functional iPSC-cardiomyocytes. As ongoing research during the COVID pandemic, Samelson et al. [128] utilized CRISPRi to identify bromodomain-containing protein 2 (BRD2) as necessary in angiotensin-converting enzyme (ACE2) transcription in cardiomyocytes, and therefore potentially a good therapeutic target for COVID. Jiang et al. [129] utilized CRISPRa to reprogram fibroblasts into cardiac progenitor cells for implantation into infarct regions of the heart towards regenerative therapeutics. More frequently used in neurodevelopmental applications, CRISPRi/a have also significantly improved the usage of iPSCs for cardiac modelling *in vitro* [2].

**Table 3:** Gene modulation with CRISPRi/CRISPRa in cardiac applications in vitro and in vivo

		Reference	Gene target
CRISPRi	in vitro (human iPSC-CMs)	CRISPRi-based system scalable towards screening for developmental pathways and disease modelling, including ion channel related disorders [130]	*NANOG, OCT4, SOX2, ROCK1, GSK3-β, MESP1, BAG3, MYBPC3, KCNH2*
		CRISPRi-based rescue of CALM2 for treatment of LQTS [131]	*CALM2*
		CRISPRi knockdown of *MESP1* to understand its role in early cardiac differentiation [124]	*MESP1*
		CRISPRi knockdown of integrin α5 subunit to understand its contribution to early mesoderm development [125]	*I*α*5 subunit*
		CRISPRi in post-differentiated hiPSC-CMs to suppress key cardiac ion channels, combined with all-optical electrophysiology [132]	*KCNH2, KCNJ2, GJA1*
		Development of a CRISPRi hiPSC line and validation of pluripotency and capability to differentiate into the three germ layers [126]	*KLF15*
		CRISPRi screen to identify druggable targets of SARS-Cov-2 in cardiomyocytes [128]	*BRD2*
	in vivo	–	*–*
CRISPRa	in vitro (human iPSC-CMs)	Generation and characterization of CRISPRa hiPSC line for disease modelling applications [127]	*KLF15*
		Reprogramming of fibroblasts to cardiac progenitors cells by CRISPRa and their successful implantation into myocardial infarction mouse models [129]	*GATA4, NKX2.5, TBX5*
	in vivo	CRISPRa mediated activation of Mef2d and Klf15 in the post-natal heart [133]	*Mef2d, Klf15*

CRISPRi/a methods have also provided new opportunities to study cardiac disease pathogenesis and to develop better treatments in an otherwise difficult to study field. Mandegar et al. [130] were the first to develop an inducible CRISPRi platform in human iPSCs and follow up RNAseq to show that it outperformed CRISPR with an active Cas9, in addition to offering reversible gene modulation. Proximity to the transcription start site (TSS) in designing gRNAs was good efficiency predictor. In addition to showing utility and specificity of CRISPRi knockdown of genes implicated in cardiac cell differentiation and illustrating temporary gene modulation of exogenous targets (e.g. calcium sensor GCaMP), they also found expected phenotypic consequences (action potential prolongation) of CRISPRi reduction of the HERG potassium ion channel in the hiPSC-CMs [130]. Limpitikul et al. [131] showcased a new therapy for personalized medicine: CRISPRi in human iPSC-CMs with gRNAs targeting calmodulin (CALM) mutations associated with long-QT syndrome corrected the action potential prolongation due to excess calcium release. Han et al. [132] developed a scalable CRISPRi platform for gene perturbation combined with optogenetics-based characterization methods (all-optical electrophysiology). They demonstrated correlative results of mRNA perturbation by CRISPRi on key cardiac ion channels and electrophysiological functional effects within the same samples. Overall, with the combined use of CRISPRi/a and patient-derived iPSCs, the technologies help efforts towards personalized medicine and patient-specific treatments.

These advancements have facilitated translational studies in vivo as well, Table 3. For example, recently Schoger et al. [133] used CRISPRa to activate *Mef2d* and *Klf15*, transcription factors controlling cardiac hypertrophy and homeostasis, as proof-of-concept deployment of CRISPRi/a to control endogenous gene transcription in the heart. In vivo neuroscience applications have advanced faster. For example, Lau et al. [134] utilized AAVs to systemically deliver CRISPRi/a tools in the mouse brain for targeted endogenous gene interference and activation. Colasante et al. [135]. reduced seizures by upregulating the potassium channel gene (*Kcna1*), demonstrating a CRISPRa-based approach to treat epilepsy. Gemberling et al. [136] developed a Cre-inducible CRISPRi/a transgenic mouse model for controlled regulation of target genes in the liver, T cells, fibroblasts, and neurons. Further in vivo developments and potential clinical translation of the CRISPRi/a tools for cardiac use face similar challenges as the other CRISPR-related methods due to packaging and difficulties achieving efficient delivery/expression of the relatively large constructs. Optimization of these tools, including quick gRNA selection for effective gene inhibition/activation, can speed up in vivo therapeutic use.

## CRISPRi/a Screens and Functional Genomics in iPSC-CMs

CRISPR screens are a powerful platform for genome-wide and high-throughput genetic exploration to probe genes, pathways, and mechanisms for biological discovery [137]. Compared to traditional RNAi libraries for loss-of-function studies, CRISPR and gRNA provide a richer set of approaches for transcriptional inhibition, activation, knockout studies on a larger set of genes. Interrogating upwards of thousands of genes to identify those critical to biological pathways and potential drug targets is possible in cardiac, neurological, metabolic diseases, cancer, immunology, and other fields. Figure 2 outlines the progression of genome-scale CRISPRi/a-mediated screens over the last decade. Gilbert et al. [138] published the first genome-scale screens for CRISPRi and CRISPRa studies. Various efforts have focused on optimizing genome-wide gRNA libraries [139–141]. Screening methods have allowed the more efficient identification of essential genes for cell survival [142], drug resistance [86, 143], protein folding [144] and iPSC differentiation [145–147], Fig. 2, Table 4. More recent studies have extended screening methods to include epigenomic, base editing, and prime editing technologies [43, 148, 149]. A particularly powerful approach to dissect the contribution of individual genes to function has been the combination of CRISPRi/a with Pertub-seq [144, 150], including analysis done at the single-cell level [151] and at genome-scale [152]. This method, which characterizes the full transcriptomics response to a single gene perturbation, is promising for deriving gene regulatory networks, GRNs for reliable predictions [150].

**Table 4:** CRISPRi/CRISPRa-based screens using human iPSCs for cardiac applications

Application	CRISPRi/CRISPRa	Application	Major Findings
iPSC	CRISPRi	Cell growth	CRISPRi-based pooled screening to identify genes for robust cellular growth in iPSCs [142]
	CRISPRi/CRISPRa	Cardiac development	Protocol outlining CRISPR based screening approaches in hiPSC to identify essential genes in CM differentiation [153]
Cardiac applications	Review	Cardiovascular disease	Review summarizing progress of identifying pathogenic variants for cardiac disease using CRISPR-edited iPSCs [154]
	Cas9	Cardiotoxicity	CRISPR/Cas9-based screening in hiPSC-CMs to explore mechanisms of doxorubicin induced cardiotoxicity [155]

**Fig. 3:**
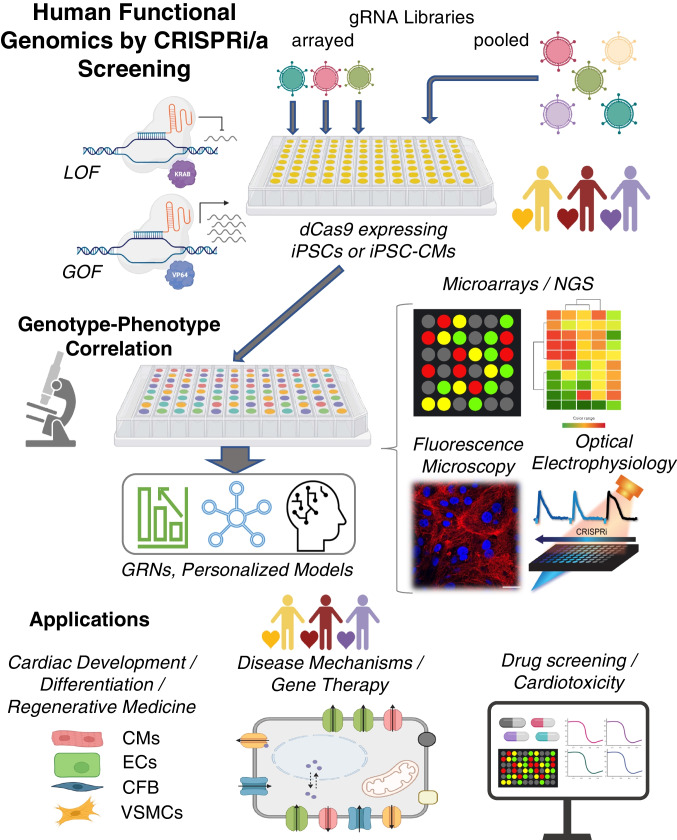
Human functional genomics by CRISPRi/a. CRISPR-based screening approaches, iPSC technology, and all-optical electrophysiology provide key elements for a high-throughput platform to perturb gene function and analyze respective genomic, protein, and functional changes for biological discovery of cardiac development and disease. Based on combined functional and transcriptomics data, one can build gene regulatory networks, GRNs, using machine learning techniques. The technology and the derived GRN models can be applied to cardiac development, guiding cell differentiation and maturation for regenerative medicine; disease modeling, drug development and cardiotoxicity testing. Biorender was used for parts of this figure

CRISPR screens fall into one of two types: pooled vs. arrayed screens, Fig. 3. When combined with differentiated human cells, such as iPSC-CMs, these screens represent a patient-specific tool towards functional genomics, i.e. identification of the contributory role of each gene to the biology of the cells/tissues studied. Scalable readouts are provided by next-generation sequencing (NGS) methods and other scalable technologies for phenotypic characterization. Such integrated tools have not been available previously for human studies and they hold a lot of promise in informing and accelerating therapeutic developments.

Pooled CRISPRi/a screens are simple to apply – mixed gRNAs for all target genes are added to the samples at once. These screens are most suitable for well stratified phenotype readouts—two or more types of responses. The simplest readouts are cell viability/proliferation assays or fluorescence-activated cell sorting (FACS) analysis. Upon presentation of a pooled CRISPRi library, cell enrichment informs mechanistic studies of cell survival and proliferation, e.g. CRISPRi-based pooled screening helped identify genes critical for cell growth in iPSCs [145, 156]. This technique is generalizable to biological outcomes beyond survival, by sorting or enriching cells with attributes of interest. Cell lines have also been generated to express a fluorescent protein when a signaling pathway is activated, which then can be processed for cell enrichment [157]. Spatial imaging, combined with labeling by antibodies, small molecules, or genetically encoded reporters can help monitor cellular activity of the screened cells. Quantification of mRNA and protein can be performed through fluorescence in situ hybridization (FISH) or flow cytometry. Single-cell RNA sequencing offers high-dimensional readouts of pooled CRISPR screens and capture of biological phenotypes not easily measurable by a single marker gene.

In contrast, arrayed CRISPRi/a screens typically present one gRNA or perturbation per target cell/sample, thus a gene activation or inhibition can be linked to complex functional responses across samples. They can be coupled to high-content screening assays for derivation of a relationship between genotype perturbation and more nuanced cellular phenotypes. The readouts can be complex—proteomics, metabolomics and functional imaging. More sophisticated cell/tissue models can be coupled with arrayed CRISPRi/a screens, e.g. 3D organoids. Overall, pooled screens enable discovery, whereas arrayed screens are better suited for validation and in-depth mechanistic studies. Arrayed gRNA screens, can be combined with high-throughput all-optical electrophysiological studies [158–160] for human functional genomics investigation of cardiac development or disease Fig. 3.

With any genetic modulation method, proper introduction of CRISPR tools and successful gene perturbation should be tested. Delivery of CRISPR tools using plasmids, mRNA, protein, or lentiviral vectors can be challenging in terminally differential cells, such as cardiomyocytes. Transduction by viral particles or lipofection and electroporation methods need to be optimized for each cell type and model organism. Although commercial gRNA libraries are available, a panel of gRNAs targeting different loci of each gene need to be evaluated to identify the efficiency of perturbation, e.g. by qPCR, which can be quite tedious or by newer sequencing methods [72]. Additionally, confirmation of each gene modulation can be done at the protein level using western blots or flow cytometry. Sometimes these methods are not well suited for the small sample size in high-throughput plates and newer developments are needed [161]. In pooled screens, cells can be evaluated strictly for cell survival and proliferation or can be selected under biological pressures such as drug treatment or viral infection, followed by more in-depth functional assays. Modular systems such as chemically- [130, 162, 163] and optically-inducible [164–166] Cas9/dCas9 allow exploration of essential genes over time. Optical control can also provide fast and precise spatial–temporal gene modulation [167]. Establishing reliable CRISPR screens offers a powerful approach to functional biology.

## Conclusions

In the last decade, we are witnessing the convergence of several scalable technologies: 1) human iPSC-derived cells with infinite renewal capacity; 2) next-generation sequencing and single cell transcriptomics; 3) optogenetics-enabled all-optical functional assays; 4) big data handling capacity, powerful and fast machine learning algorithms; and 5) the CRISPR-inspired and CRISPR-derived gene modulation techniques, discussed here. The full seamless integration of these is yet to come, but they enable progress towards human functional genomics, Fig. 3. The combination of these tools allows for unprecedented look at the role of each gene in shaping human biological responses in health and disease; they can help uncover intricate systems-level interactions of genes leading to a particular phenotype. More precise gene modulation techniques, free of off-target effects, provide a critical perturbation tool to dissect such relationships. Advancements in human iPSC technology offer a wider representation of demographics in understanding human biology and its nuances. Patient-specific testing, with direct translational value is becoming possible. Capturing complex phenotypes, which accompany most disease conditions, is facilitated by advancements in transcriptomics and contactless methods of functional characterization beyond live/dead assessment. The sheer volume of high-content data generated from the convergence of these technologies presses the need for better computational tools and learning algorithms. The comprehensive characterization of responses can enable the generation of “digital twins” (virtual models) for patients in the context of drug application and clinical decision making.

The translational impact of this convergence of techniques is seen in informing drug development, cardiotoxicity testing, regenerative medicine and gene therapy. Cardiology is one of the fields most directly benefiting from these approaches, due to the complex nature of functional responses and the need for human experimental models. CRISPR-based gene modulation methods have already seen in vivo use, and some – clinical translation. Previously non-treatable genetic disorders can be tackled, with hopefully fewer side effects, compared to traditional pharmacology. Shared challenges for the in vitro and the in vivo deployment of the gene modulation techniques concern effective and safe delivery methods. Further investment in viral and non-viral delivery approaches to gene modification is needed, as these are at the heart of faster translation of gene therapy in the clinic.

## Data Availability

No new data were generated for this review.
